# Metformin exerts antitumor activity via induction of multiple death pathways in tumor cells and activation of a protective immune response

**DOI:** 10.18632/oncotarget.25380

**Published:** 2018-05-25

**Authors:** Felipe V. Pereira, Amanda Campelo L. Melo, Jun Siong Low, Íris Arantes de Castro, Tárcio T. Braga, Danilo C. Almeida, Ana Gabriela U. Batista de Lima, Meire I. Hiyane, Matheus Correa-Costa, Vinicius Andrade-Oliveira, Clarice S.T. Origassa, Rosana M. Pereira, Susan M. Kaech, Elaine G. Rodrigues, Niels Olsen S. Câmara

**Affiliations:** ^1^ Laboratory of Transplantation Immunobiology, Department of Immunology, University of São Paulo, Institute of Biomedical Sciences, 05508-900 São Paulo, SP, Brazil; ^2^ Laboratory of Cancer Immunobiology, Department of Microbiology, Immunology and Parasitology, Escola Paulista de Medicina, Universidade Federal de São Paulo (EPM-UNIFESP), 04023-062 São Paulo, SP, Brazil; ^3^ Laboratory of Infectious Diseases, Department of Immunology, Institute of Biomedical Sciences, University of São Paulo, 05508-900 São Paulo, SP, Brazil; ^4^ Department of Immunobiology, Yale University School of Medicine, 06520 New Haven, CT, USA

**Keywords:** metformin, tumor immunity, T cells, cell death, sitagliptin

## Abstract

The antitumor effect of metformin has been demonstrated in several types of cancer; however, the mechanisms involved are incompletely understood. In this study, we showed that metformin acts directly on melanoma cells as well as on the tumor microenvironment, particularly in the context of the immune response. *In vitro*, metformin induces a complex interplay between apoptosis and autophagy in melanoma cells. The anti-metastatic activity of metformin *in vivo* was assessed in several mouse models challenged with B16F10 cells. Metformin's activity was, in part, immune system-dependent, whereas its antitumor properties were abrogated in immunodeficient (NSG) mice. Metformin treatment increased the number of lung CD8-effector-memory T and CD4^+^Foxp3^+^IL-10^+^ T cells in B16F10-transplanted mice. It also decreased the levels of Gr-1^+^CD11b^+^ and RORγ^+^ IL17^+^CD4^+^ cells in B16F10-injected mice and the anti-metastatic effect was impaired in RAG-1^−/−^ mice challenged with B16F10 cells, suggesting an important role for T cells in the protection induced by metformin. Finally, metformin in combination with the clinical metabolic agents rapamycin and sitagliptin showed a higher antitumor effect. The metformin/sitagliptin combination was effective in a BRAFV600E/PTEN tamoxifen-inducible murine melanoma model. Taken together, these results suggest that metformin has a pronounced effect on melanoma cells, including the induction of a strong protective immune response in the tumor microenvironment, leading to tumor growth control, and the combination with other metabolic agents may increase this effect.

## INTRODUCTION

Melanoma is the most aggressive form of dermal/skin cancer. Its development is associated with both genetic predisposition and exposure to environmental factors [1]. Although great advances have been achieved in melanoma treatment, including the recent introduction of targeted therapies such as vemurafenib, a BRAF inhibitor, and ipilimumab, an anti-CTLA-4 agent, new drugs and therapeutic strategies are urgently needed [2, 3].

Metformin is one of the most commonly prescribed drugs for the treatment of type 2 diabetes (T2D). Its anti-diabetic effect is attributed to inhibition of hepatic gluconeogenesis, which is possibly associated with insulin-mediated increase in glucose uptake in skeletal muscle [4]. Recently, epidemiological data and experimental studies have shown the antitumor effect of metformin against several types of cancer, including melanoma [5, 6]. Metformin can reduce levels of Ki-67, a marker of proliferation, in biopsy samples of non-diabetic women with breast cancer [7]. However, the mechanisms by which metformin acts to attenuate tumor progression are not well-defined, and relevant information is limited. Assessing the *in vitro* anti-proliferative properties of metformin in patients is challenging, especially when it is effective only at supra-physiological concentrations *in vivo* [8]. The systemic effects of metformin on tumorigenesis are associated with decreased hyperinsulinemia, which in turn is associated with poor prognosis in several types of cancer, including breast, colon, and prostate [9]. Additional studies have shown that metformin can influence cancer cells directly, mainly via AMP-activated kinase (AMPK)-dependent and independent mechanisms [10].

Moreover, it has been reported that metformin can affect the immune system in healthy patients and in disorders such as autoimmune disease, tuberculosis, and cancer [11–14]. Some studies have also demonstrated that metformin affects T effector cell subsets and promotes the generation of memory T cells via the AMPK pathway [15–17]. However, it has also been suggested that metformin can regulate cell growth and T cell proliferation via mechanisms that are not dependent on AMPK expression [18]. Metformin affects lymphocytes, macrophages, neutrophils, and other immune cells, and can modulate the secretion of a number of cytokines, such as interleukin (IL)-10, IL-17, IFN-γ, IL-22, and IL-6 [14, 19–21].

In this study, we tested the hypothesis that metformin could act bidirectionally on melanoma cells as well as on effector protective immune cells, contributing to tumor control. We evaluated multiple mechanisms of cell death in melanoma cells, including apoptosis, autophagy, caspase-independent pathways, and the participation of the receptor-interacting serine/threonine-protein kinase 1 (RIPK1) cascade. We tested the anti-metastatic effect of metformin in a set of B16F10-challenged mouse models to evaluate the role of the immune system in metformin's protective action. The combined effects of metformin with rapamycin and sitagliptin were also evaluated. Collectively, these findings indicate that the anticancer actions of metformin are multi-faceted.

## RESULTS

### Metformin affects melanoma cells and migration

**Figure 1:**
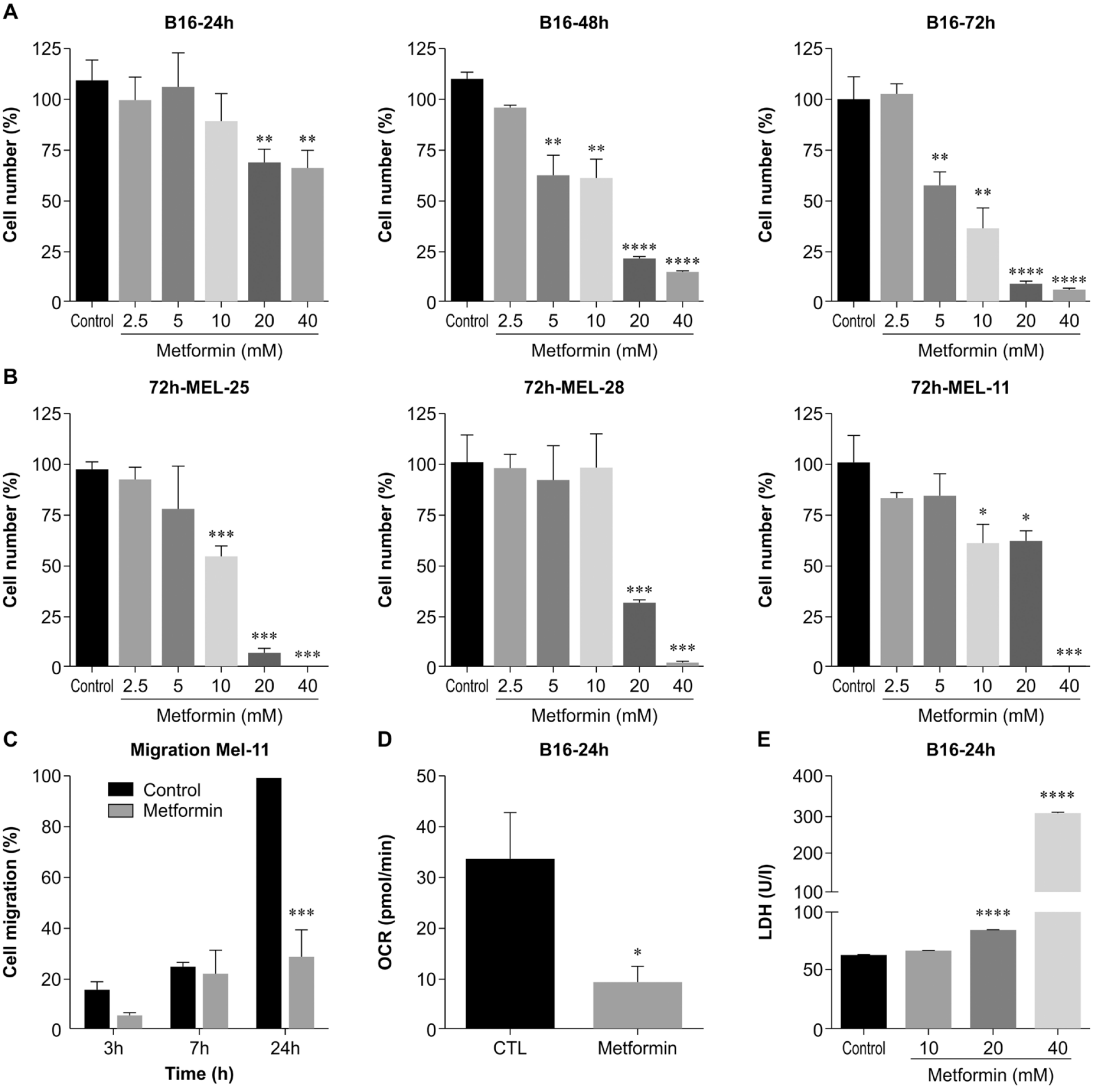
Metformin effects in melanoma cells *in vitro* **(A)** B16F10 cells (5×10^3^) were incubated for 24, 48, or 72 h with metformin (0–40 mM), and cell viability was measured using the MTT assay (^**^p<0.002, ^****^p<0.0001). **(B)** Different patient melanoma cells (Mel25, Mel28, and Mel11) were incubated with metformin (0–40 mM) for 72 h, and cell viability was measured using the MTT assay (^***^p<0.003). **(C)** Patient Mel11 cell migration was determined by measuring wound width at 0, 3, and 7 h, and 24 h after incubation with 5 mM of metformin (^***^p<0.003). **(D)** Oxygen consumption rate of B16F10 cells treated with metformin (10 mM) for 18 h (^*^p<0.05). **(E)** B16F10 cells were incubated for 24 h with metformin (0–40 mM), and lactate dehydrogenase (LDH) levels were measured in the supernatant after a 24-h incubation (^****^p<0.0001).

To evaluate the direct effect of metformin on melanoma cells, we performed *in vitro* viability assays where dose- and time-dependent effects on B16F10 murine melanoma cells were observed. Treatment with different concentrations of metformin for 24, 48, and 72 h reduced B16F10 cell viability (Figure 1A). Interestingly, human melanoma cells isolated from patients were also sensitive to metformin. MEL25, MEL28, and MEL11 human cell lines were treated for 72 h with different concentrations of metformin (0–40 mM), and cell viability was assessed by the MTT assay (Figure 1B). MEL25 was the most metformin-sensitive cell line, whereas MEL28 cells exhibited marked resistance to treatment, and MEL11 showed intermediate sensitivity (Figure 1B). In all three cases analyzed, the effect of metformin treatment was dose-dependent.

The ability of metformin to modulate cell migration was tested using a monolayer wound-healing assay. MEL11 human melanoma cells were incubated with metformin, at a concentration (5 mM) that did not induce cell death, for 24 h. This treatment inhibited cell migration compared to that of control cells (Figure 1C; Supplementary Figure 1). Metformin had no effect on the migration of MEL25 and MEL28 cell lines (data not shown).

Several studies have reported that metformin acts on mitochondria through inhibition of the respiratory complex I and alteration of energy production by tumor cells [22–23]. We therefore measured the oxygen consumption rate (OCR) of B16F10 cells after treatment with metformin. We observed that metformin treatment reduced OCR in treated cells compared with that in untreated cells (Figure 1D).

This dose-dependent effect of metformin on cell viability was also evident in the assay for the determination of lactate dehydrogenase (LDH) release; treatment for only 24 h with 20 and 40 mM metformin markedly increased LDH release in the culture media (Figure 1E).

### Metformin induces different mechanisms of cell death in melanoma cells

**Figure 2:**
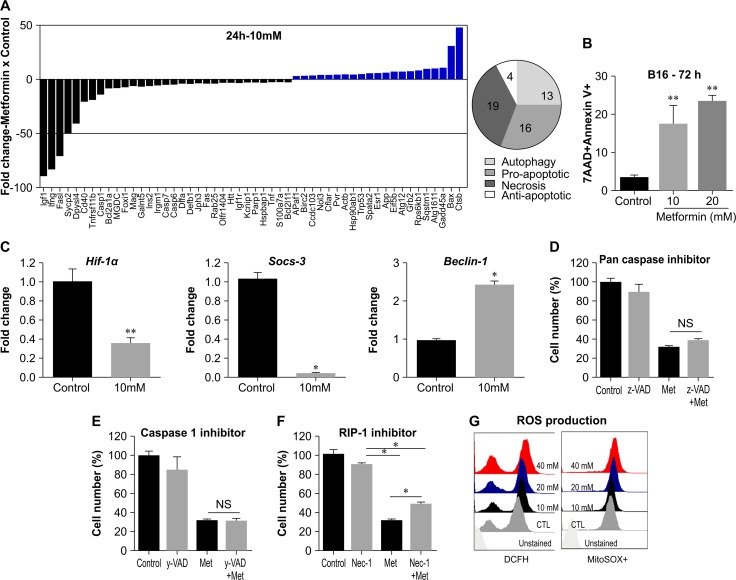
Metformin induces different cell death mechanisms in B16F10 melanoma cells **(A)** Fold increase (>2) or decrease (<2) in gene expression after B16F10 cells were treated for 24 h with metformin (10 mM), measured using quantitative RT-PCR. Pie chart shows the distribution of genes related to a specific cell death process. **(B)** B16F10 cells treated for 72 h with metformin (10 Mm) were stained with annexin V and 7-AAD. **(C)** mRNA transcript quantification of HIF-1α, Socs-3, and Beclin-1 in B16F10 cells treated for 24 h with metformin (10 mM) (^**^p<0.001). **(D)** B16F10 cells treated for 24 h with metformin (20 mM) in the presence of Z-VAD-FMK (20 μM), **(E)** Z-YVAD-FMK (20 μM), **(F)** necrostatin-1 (Nec-1, 100 μM). Cell viability was measured using the MTT assay. **(G)** B16F10 cells were incubated for 24 h with metformin (0–40 mM). Representative histograms and graphs of flow cytometry analyses showing mean fluorescent intensity of DCFH (left) or oxidized MitoSOX (right) following treatment.

To gain more insight into the pathways by which metformin impairs cell viability, we performed a focused gene pathway assay comprising the gene expression profile of key molecules associated with central mechanisms of cell death (i.e. apoptosis, autophagy, and necrosis). Genes that showed at least a two-fold difference in expression between metformin-treated cells (10 mM, 24 h) and controls (RPMI media only) were plotted (Figure 2A). Metformin treatment greatly increased the mRNA expression of *Ctsb and Bax*, whereas levels of *Igf1*, *Ifng*, *Fasl*, and *Sycp2* transcripts markedly decreased (Figure 2A, bar graph). Overall, metformin modulated the genes associated with various death processes as follows: autophagy (13 genes), pro-apoptosis (16 genes), necrosis (19 genes), and anti-apoptosis (4 genes) (Figure 2A, pie chart). We also found an increase in B16F10 apoptosis using Annexin V/7-AAD labeling after treatment with metformin (Figure 2B). Metformin treatment decreased the gene expression of *Hif-1α* and *Socs-3*, genes associated with cancer cell resistance to death [24, 25], in B16F10 cells (Figure 2C); these genes have been reported as targets of metformin in different tumor types [25, 26]. Conversely, the expression of *Beclin1*, an autophagy-related gene, increased after metformin treatment (Figure 2C).

The expression of *Casp1, Casp6*, and *Casp7* was reduced by metformin treatment, as demonstrated by the PCR array assay (Figure 1A, bar chart). To assess whether caspases are directly involved in the cell death process mediated by metformin, we treated B16F10 cells with metformin (20 mM) for 24 h in the presence of either a pan-caspase inhibitor (Z-VAD-FMK) or a caspase 1 inhibitor (Z-YVAD-FMK). Neither inhibitor affected metformin action (Figure 2D–2E), suggesting that metformin acts through a caspase-independent mechanism in the B16F10 melanoma cell line.

Necroptosis is a process of programmed cell death that acts independently of caspase activity and incorporates characteristics of both necrosis and apoptosis. RIPK1, RIPK3, and mixed-lineage kinase domain-like protein (MLKL) are receptor-interacting proteins that play a central role in the formation of the necrosome, a molecular structure that results in initiation of cell death [27]. In this regard, we assessed the viability of B16F10 cells treated with metformin in the presence of an RIPK1 inhibitor (necrostatin-1). The effect of metformin was partially inhibited by necrostatin-1 (Figure 2F). These findings suggest that RIPK1 may participate in the cell death process induced by metformin in B16F10.

The generation of reactive oxygen species (ROS) is an intracellular effect that can occur in different cell death processes, including apoptosis, necrosis, necroptosis, and even autophagy [28]. Detection of intracellular and mitochondrial ROS in B16F10 was performed by flow cytometry using DCFH and MitoSox probes, respectively (Figure 2G). A dose-dependent increase was observed in intracellular (DCFH, left) and mitochondrial (MitoSox, right) ROS levels after treatment with metformin, compared with untreated cells (Figure 2G). Altogether, these data show that the effect of metformin on B16F10 cells involves multiple combined mechanisms associated with cell death.

### Anti-metastatic effects of metformin require an intact immune system

**Figure 3:**
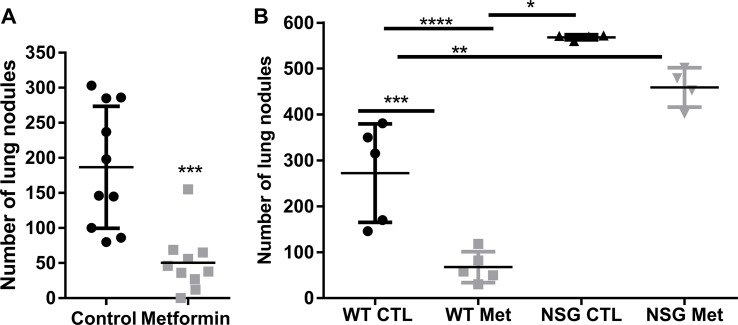
Anti-metastatic activity of metformin requires an intact immune system **(A)** C57BL/6 mice were intravenously challenged with B16F10 cells, and treated by gavage with 500 mg/kg metformin or phosphate-buffered saline (PBS) daily from day 3 after challenge. Quantification of the number of lung nodules in each group, n=12, ^***^p<0.0002, mean ± standard deviation. **(B)** B16F10 cells (5×10^5^) were injected intravenously into C57BL/6 mice and NOD-SCID gamma null (NSG) mice. Three days after the challenge, the animals were treated with metformin (500 mg/kg) or PBS daily by gavage. Pulmonary nodules were counted on day 15. n=5 animals per group (^*^p<0.05, ^**^p<0.001, ^***^p<0.0001, ^****^p<0.001).

To evaluate the anti-metastatic effects of metformin, C57BL/6 mice were inoculated intravenously with B16F10 murine melanoma cells and treated daily with metformin or vehicle for 12 days, starting 3 days after tumor cell challenge. The number of lung nodules was counted 24 h after the last dose of metformin. Oral administration of metformin significantly reduced the total number of lung metastases compared with control vehicle-treated mice (Figure 3A). Interestingly, we did not observe a protective effect of metformin in B16F10 melanoma cells inoculated subcutaneously (Supplementary Figure 2). Although metformin exerted a direct effect on melanoma cells, its influence on the tumor microenvironment *in vivo* and on the immune system has not been elucidated. To evaluate the role of immune effector function in metformin-mediated reduction in B16F10 lung metastasis, we analyzed B16F10 tumor development in metformin-treated severely immunodeficient NSG mice. Corroborating our previous result, metformin treatment significantly reduced the number of lung metastases in C57BL/6 mice compared with metastases in untreated control mice (Figure 3B). Notably, the NSG mice group showed a significantly increased number of pulmonary nodules in comparison with C57BL/6 mice, and metformin-treated NSG mice presented a no significant difference in the number of lung nodules when compared with PBS-treated NSG mice (Figure 3B). These data suggest that the anti-metastatic effect of metformin is, at least in part, mediated by the immune system.

### Metformin treatment modulates local and systemic immune response

**Figure 4:**
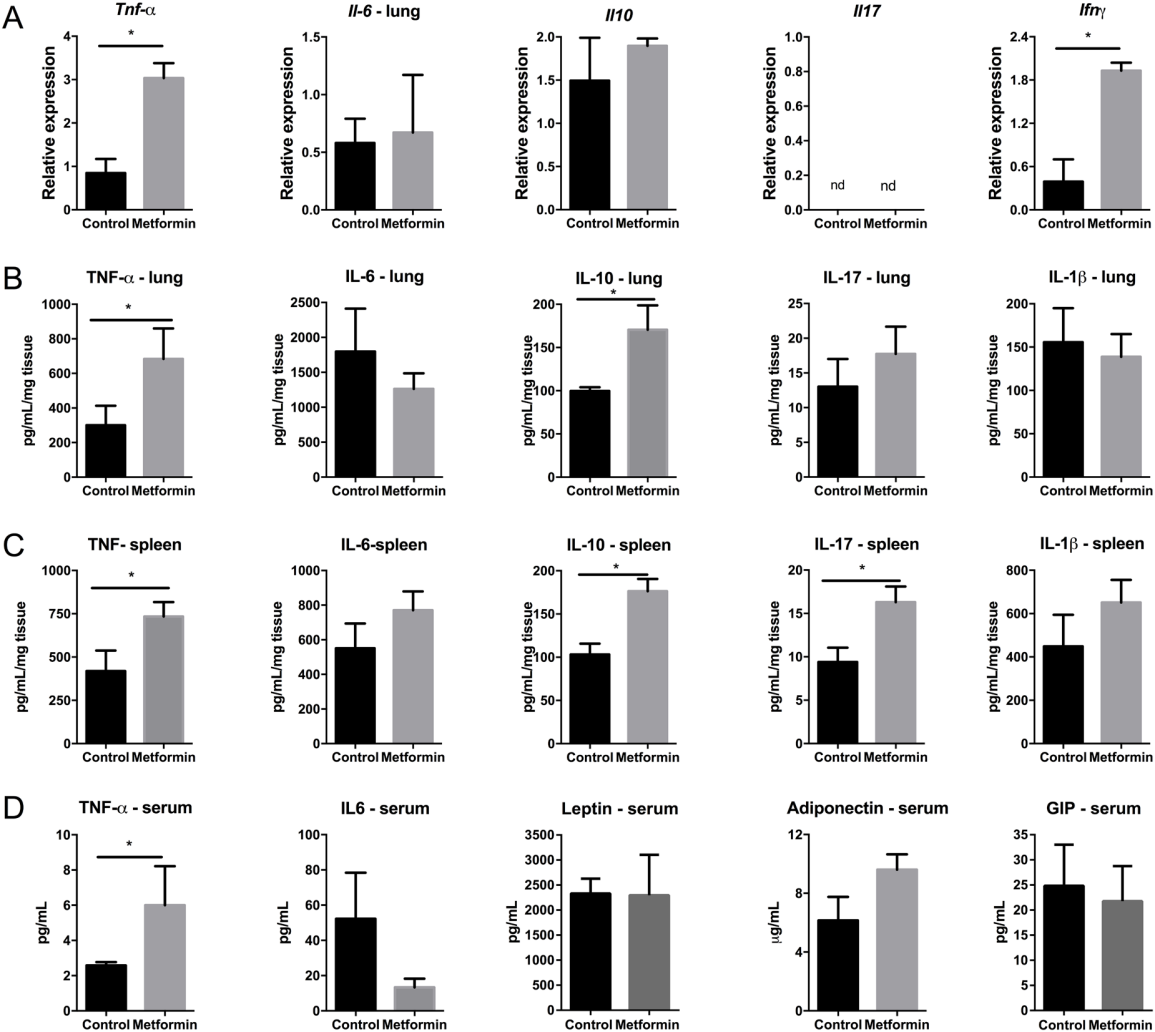
Tumor microenvironment and systemic profile of cytokines and hormones in mice treated with metformin **(A)** Cytokine (IFN-γ, TNF-α, IL-10, IL-6, and IL-17) mRNA expression was determined in the lungs of C57BL/6 mice challenged intravenously with B16F10 cells and treated with metformin. Values are expressed relative to a control group. The cycle thresholds (Ct) for the gene and the internal control were determined for each sample. The relative mRNA expression was calculated as 2-ΔΔCT relative to HPRT. Levels of the same cytokines were determined in the lungs **(B)** and spleen **(C)** of these animals by ELISA. **(D)** TNF-α, IL-6, leptin, adiponectin, and GIP were measured in the sera of these mice by ELISA. n=5; values are expressed as mean ± standard error. ^*^p<0.05. Images are representative of at least two independent assays.

To elucidate the immune pathways that contribute to the protective effect of metformin in metastatic melanoma, firstly we evaluated gene expression and protein levels of different cytokines in the lung tumor microenvironment. We observed an increased expression of *Ifnγ* and *Tnfα* genes in the lungs of metformin-treated mice compared with expression in the control group (Figure 4A). In contrast, *Il10* and *Il6* gene expression was unaffected by metformin compared with expression in the control group. *Il17* gene expression was not detected in either group (Figure 4A). Protein levels of cytokines were measured by ELISA in lung homogenates on the 15^th^ day after B16F10 cell inoculation. There was a significant increase in the levels of TNF-α and IL-10, but no change in IL-1β, IL-6, and IL-17 expression in the lungs of the metformin-treated group compared with expression in the control group (Figure 4B).

In addition to a local immune response, metformin treatment of B16F10-challenged mice induced a systemic antitumor response. Metformin-treated mice exhibited significantly increased TNF-α, IL-10 and IL-17 concentrations in splenocyte homogenates compared with concentrations in the control group, whereas IL-1β and IL-6 levels were similar in treated and untreated mice (Figure 4C). The levels of TNF-α significantly increased in the sera of the metformin-treated group compared to levels in the control group. Levels of adiponectin increased, and IL-6 levels tended to decrease, but these findings were not statistically significant (p>0.05) (Figure 4D). These results suggest that metformin modulates cytokine profiles locally and systemically in response to B16F10 challenge.

### Metformin treatment decreases Gr-1^+^CD11b^+^cells and increases T cells infiltration into tumor

**Figure 5:**
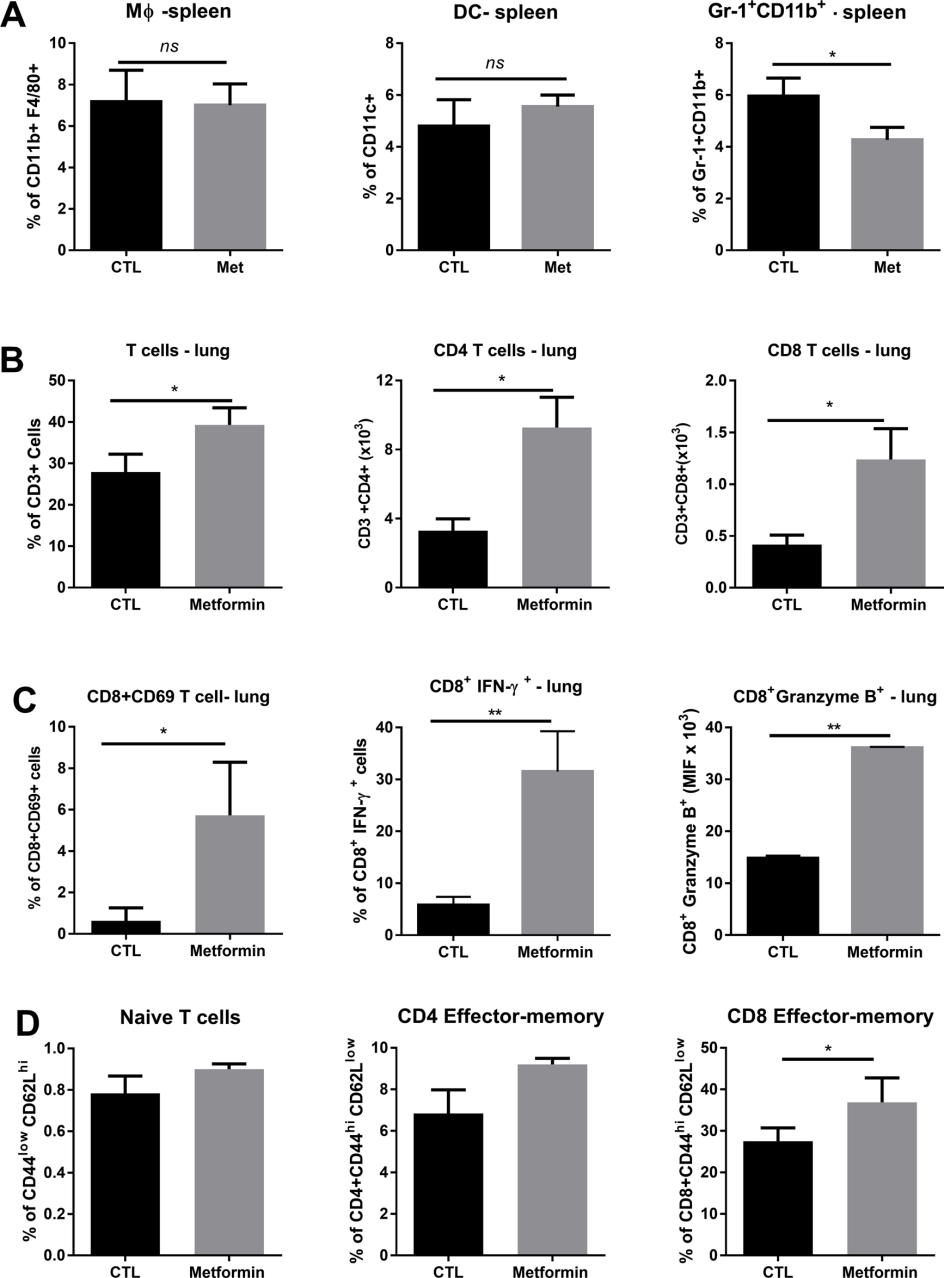
Effect of metformin on myeloid and T cells **(A)** C57BL/6 mice intravenously challenged with B16F10 cells were treated with metformin or phosphate-buffered saline (PBS) and numbers of CD11b^+^F4/80^+^, CD11c^+^ or Gr-1^+^CD11b^+^ cells were assessed in spleens by fluorescence-activated cell sorting. Percentage of T cells in the lung, absolute count of T CD4^+^ and T CD8^+^ lung cells **(B)**, percentage of CD8^+^CD69^+^, CD8^+^IFN-γ^+^, and CD8^+^granzyme B^+^ cells **(C)**. Number of naïve T cells, CD4^+^CD44^hi^CD62L^low^, and CD8^+^CD44^hi^CD62L^low^ cells **(D)**. All quantities in absolute cell number or percentage ± standard deviation. ^*^p<0.05.

We investigated whether metformin treatment affected immune cell populations involved in the antitumor effect, playing a protective role. First, the frequency of myeloid compartment cells was evaluated. Macrophages (CD11b^+^F4/80^+^) and dendritic cells (CD11c^+^) in spleens were examined 15 days after the B16F10 challenge in metformin- and vehicle-treated C57BL/6 mice; no differences were observed (Figure 5A). Metformin treatment decreased the frequency of Gr-1^+^CD11b^+^cells in the spleen, cells with a myeloid-derived suppressor (MDSC) phenotype, which impair anti-tumor responses (Figure 5A).

Next, we analyzed the T cell subsets present in the lungs of metformin-treated C57BL/6 mice by flow cytometry, 15 days after i.v. challenge. The number of CD3^+^T cells increased in the metformin-treated group compared with the control group (Figure 5B). Metformin also increased the numbers of CD4^+^ and CD8^+^ T cells, and of CD8^+^CD69^+^, CD8+IFNγ^+^, and CD8^+^Gzb^+^ T cells (Figure 5B–5C). Numbers of naïve CD3^+^ T cells (CD44^lo^CD62L^hi^) and central-memory CD4^+^ T cells (CD44^hi^CD62L^hi^) were similar between the metformin-treated and control groups (Figure 5D). However, metformin treatment significantly increased the numbers of CD3^+^ effector-memory CD8^+^ T cells (CD44^hi^CD62L^lo^) in comparison with their numbers in the control group (Figure 5D). These results suggest that T cells lung infiltration is connected to the anti-metastatic response induced by metformin.

### T cells are essential for the anti-metastatic effect of metformin

**Figure 6:**
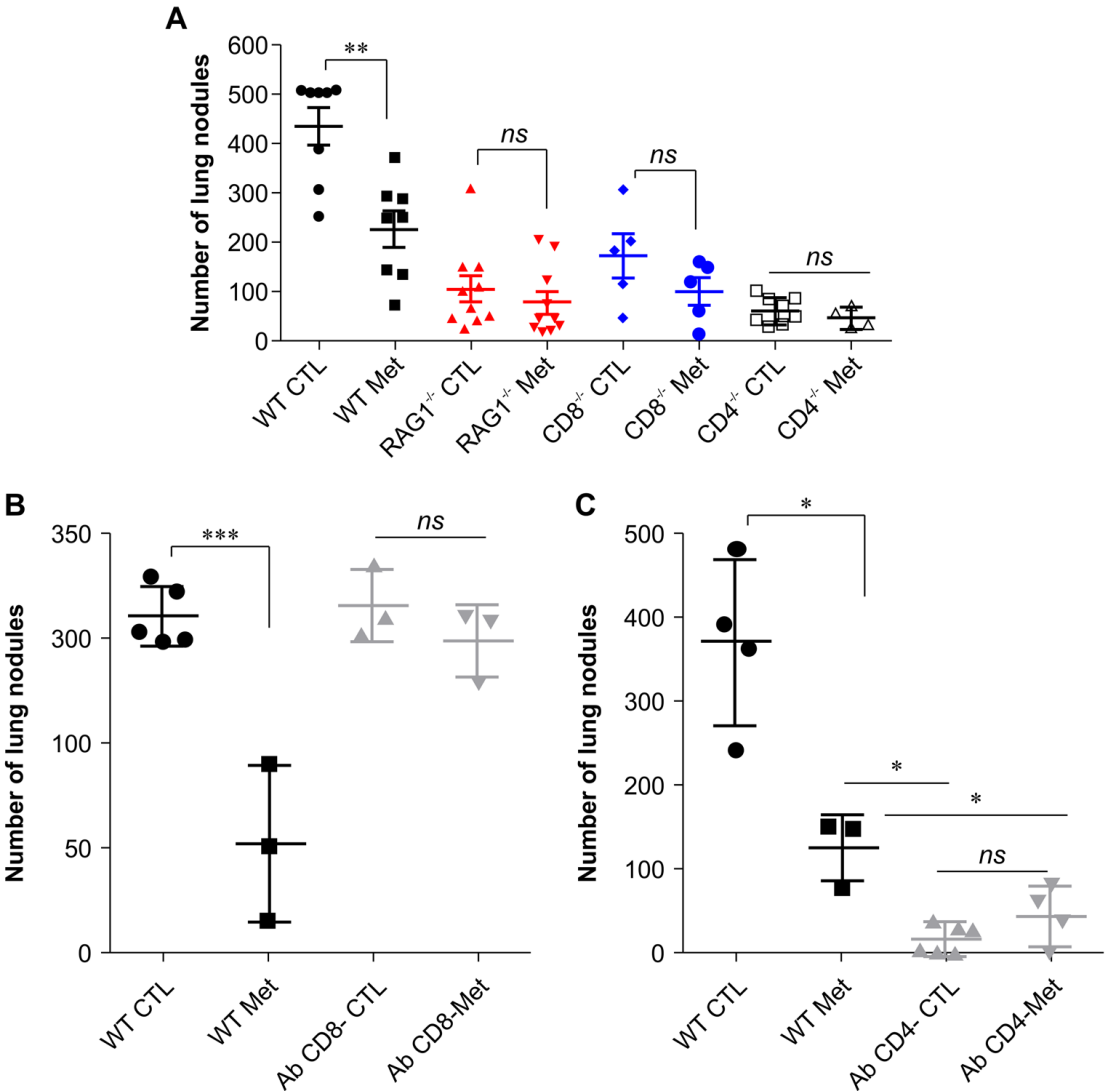
**(A)** Anti-metastatic activity of metformin with T cell participation. B16F10 cells (5×10^5^) were injected intravenously into C57BL/6, RAG^−/−^, CD4^−/−^, or CD8^−/−^ mice, and the animals were treated with metformin or phosphate-buffered saline (PBS) (n=5–11, pooling at least two experiments). ^*^p<0.05, ^**^p<0.01. **(B)** B16F10 cells (5×10^5^) were injected intravenously into C57BL/6 mice treated with metformin or PBS; the animals were administered 200 μg of an anti-CD8 antibody intraperitoneally on days 3, 7, and 11. n=4–5 mice per group (^*^p<0.05). **(C)** B16F10 cells (5×10^5^) were injected intravenously into C57BL/6 mice treated with metformin or PBS; the animals were administered 500 μg of an anti-CD4 antibody intraperitoneally on days 1 and 5. n=4–5 mice per group (^*^p<0.05). Values are expressed as mean ± standard deviation.

To functionally determine whether T cells are involved in the anti-metastatic effect of metformin, we injected B16F10 melanoma cells into C57BL/6 (WT), RAG1^−/−^, CD8^−/−^, and CD4^−/−^ mice (Figure 6A). As previously observed, metformin reduced the number of lung nodules in the immunocompetent (WT) mice compared with the number of lung nodules in the control group. Although RAG1^−/−^, CD8^−/−^ and CD4^−/−^ mice exhibited a significant reduction in the number of lung nodules compared to that in the WT group, we observed a higher lung nodules frequency in RAG1^−/−^, CD8^−/−^ and CD4^−/−^ hosts treated with metformin (75%, 60%, and 76%, respectively) compared with WT mice metformin-treated (51%), (Figure 6A). To confirm whether the effects observed in CD8^−/−^ and CD4^−/−^ mice were directly related to the absence of these cells, CD8 and CD4 cells in WT mice were depleted in each subset 1 day prior, and 5 days after i.v challenge with B16F10 cells. After depletion of CD8^+^ T cells, metformin-treated and untreated animals showed similar number of lung nodules. However, depletion of CD8^+^ T cells abrogated metformin anti-metastasis effect in the melanoma model (Figure 6B). CD4-depleted animals showed a strong reduction in the number of lung nodules compared to non-depleted animals, showing that CD4^+^ T cells are important for metastatic melanoma development. Metformin-treated, CD4-depleted mice also showed a reduction in the number of lung nodules compared to non-depleted mice (Figure 6C). These results suggest the importance of T lymphocytes in the protective response induced by metformin.

### Metformin treatment increases the population of CD4^+^Foxp3^+^IL-10^+^ cells and decreases ROR-γ^+^IL-17A^+^CD4^+^ T cells in tumor-bearing mice

**Figure 7:**
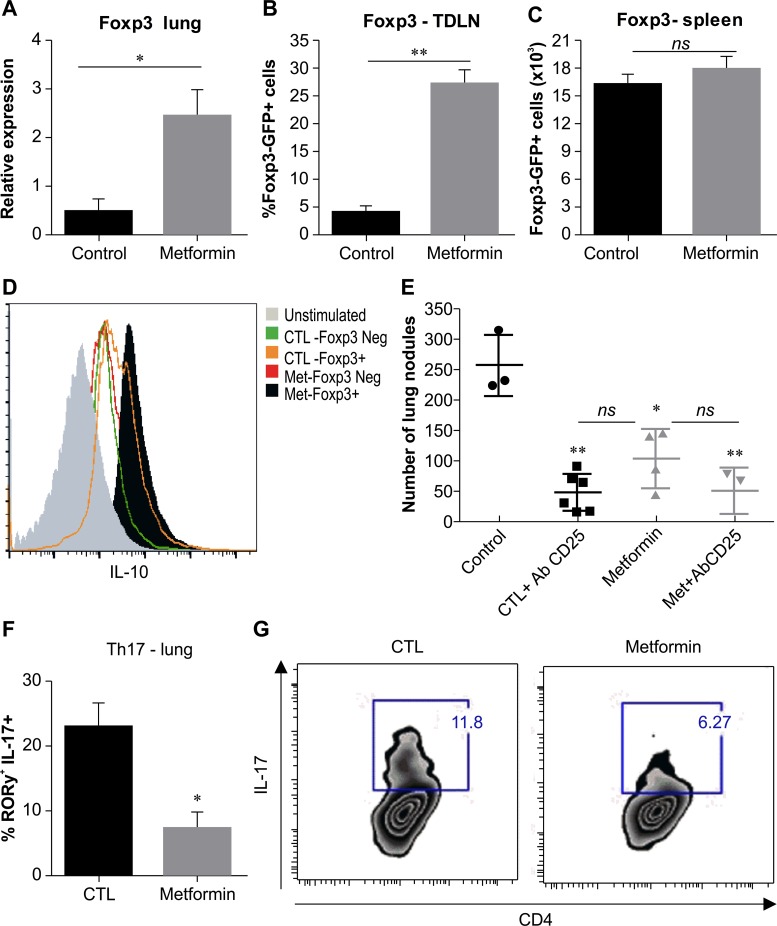
Metformin treatment induces an increase in CD4+Foxp3+IL-10+ cells *in vivo* **(A)** C57BL/6 mice were intravenously challenged with B16F10 cells, and treated by gavage with 500 mg/kg metformin or phosphate-buffered saline (PBS) daily from day 3 after challenge. Foxp3 mRNA expression in the lung tumor burden was measured by real-time PCR on day 15 after tumor challenge. Number of total Foxp3^+^ GFP^+^ cells from TDLN **(B)** or spleen **(C)** of Foxp3-gfp mice intravenously challenged with B16F10 cells and treated by gavage with metformin or phosphate-buffered saline (PBS). **(D)** Sorted Foxp3^+^ or Foxp3^−^ cells were stimulated with phorbol myristate acetate (PMA) and ionomycin (IOM) and then stained for intracellular IL-10 from the spleens of Foxp3-gfp knock-in mice challenged with B16F10 cells and treated by gavage with 500 mg/kg metformin or PBS for 15 days. **(E)** Mice were treated with intraperitoneal injection on days 1, 4, and 7 of anti-CD25 (500 μg/dose), followed by treatment with metformin (500 μg/mouse). **(F)** Number of CD4^+^RORγ^+^ T cells in the lungs of mice intravenously challenged with B16F10 cells and treated with metformin. **(G)** Naïve CD4^+^ T cells were sorted from C57BL/6J mice and cultivated in the presence of TGF-β (1 ng/mL), IL-6 (50 ng/mL), and anti-CD3/antiCD28 with or without 10 mM of metformin. Relative mRNA expression was calculated as 2-ΔΔCT relative to HPRT. n=3; values are expressed as mean ± standard error. ^*^p<0.05, ^**^p<0.01. Images are representative of at least two independent assays.

Based on our observation of a consistent increase in local and systemic IL-10 concentrations, associated with elevated numbers of CD4^+^ T cells in the lungs of metformin-treated tumor-bearing mice, we subsequently assessed whether Treg cells could be induced in this context. C57BL/6J mice were inoculated i.v. with B16F10 melanoma cells, and *Foxp3* expression in the lungs was evaluated by real-time quantitative PCR. Metformin-treated animals showed a significant increase in *Foxp3* gene expression compared with the control group (Figure 7A). We also injected B16F10 cells into *Foxp3-gfp* knock-in mice, which co-express EGFP and the regulatory T cell-specific transcription factor Foxp3 under the control of the endogenous promoter, and then treated these animals with metformin. Fifteen days after tumor challenge, the frequency of Foxp3-GFP^+^ cells in the tumor draining lymph node (TDLN) and spleen of treated and untreated animals was analyzed by fluorescence-activated cell sorting (FACS). Metformin-treated mice showed a significant increase in cell numbers in the lungs (Figure 7B), but not in the absolute numbers in the spleen (Figure 7C). To further investigate whether CD4^+^Foxp3^+^ cells induced by metformin in tumor-bearing mice were producing IL-10, CD4^+^ T cells were purified from the spleen of *Foxp3-gfp* knock-in mice and stimulated with phorbol myristate acetate (PMA) and ionomycin, followed by measurement of intracellular IL-10 levels (Figure 7D). IL-10 expression levels in CD4^+^Foxp3-GFP^−^ cells from metformin-treated and control groups were similar, but intracellular IL-10 expression in CD4^+^Foxp3-GFP^+^ cells from mice treated with metformin was higher than in the untreated-control group (Figure 7D).

To determine how these CD4^+^Foxp3^+^IL-10^+^ cells affect the anti-metastatic effects of metformin, we injected C57BL/6J mice with anti-CD25 1 day prior, and 1 and 7 days after B16F10 melanoma inoculation (Figure 7E). A reduction in the number of lung nodules was observed in CD25-depleted, melanoma-bearing untreated mice compared with non-depleted control mice. Curiously, the tumor protection induced by metformin was not altered when CD25^+^ cells were depleted, suggesting that CD4^+^Foxp3^+^IL-10^+^ T cells induced by metformin treatment do not impair the observed antitumor effect. Previous work has shown that metformin can inhibit the differentiation of Th17 cells and thereby attenuate inflammatory and autoimmune diseases [15, 29]. As shown in Figure 7F, metformin decreased the percentage of ROR-γ^+^IL-17A^+^CD4^+^ T cells in lung tumor mice. In addition, metformin diminished polarization *in vitro* of CD4^+^IL-17^+^ T cells (Figure 7G).

### Metformin combined with rapamycin or sitagliptin prevents lung metastasis

**Figure 8:**
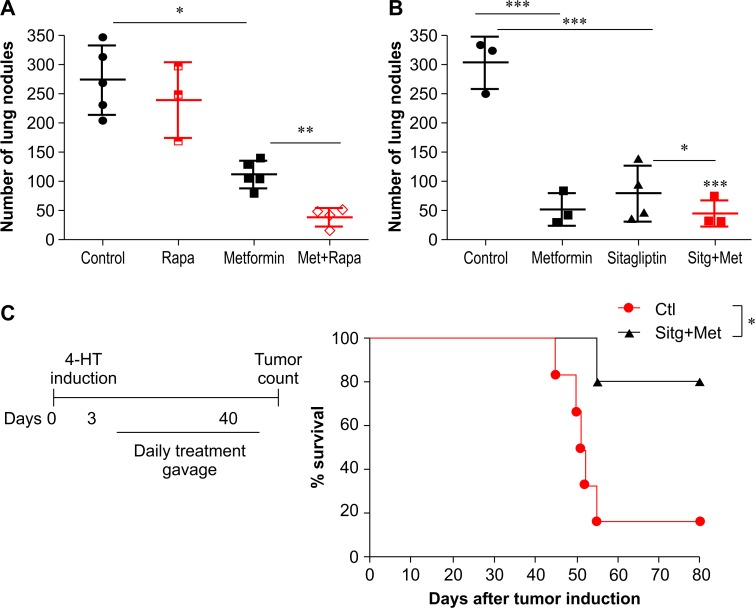
Anti-metastatic effect of metformin in combination with rapamycin or sitagliptin in a BRAF V600 murine model **(A)** C57BL/6 mice intravenously challenged with B16F10 cells (5×10^5^) were treated with phosphate-buffered saline (PBS), metformin (500 mg/kg), rapamycin (rapa, 1.5 mg/kg), or metformin + rapa, starting 3 days after challenge (n=3–5). The number of lung nodules was evaluated 15 days after the challenge. **(B)** C57BL/6 mice intravenously challenged with B16F10 cells (5×10^5^) were treated with PBS, metformin, sitagliptin (50 mg/kg), or sitagliptin/metformin starting 3 days after the challenge (n=3–4). **(C)** Kaplan-Meier survival curves comparing Tyr:CreER; BrafCA/+ and Ptenlox/lox mice treated with sitagliptin/metformin 45 days after topical administration of 4-hydroxytamoxifen (4-HT); pool of two independent assays evaluated by the log-rank test (n=7–8). Animals are represented individually; mean and standard deviation are shown by horizontal lines. ^*^p<0.05; ^**^p<0.001; ^***^p<0.0001; ns, not significant.

Finally, we investigated the effects of metformin in combination with other target agents used for cancer treatment and other pathologies such as obesity, diabetes, and neurological disorders. We first evaluated rapamycin, a drug with well-described anticancer properties that acts as a specific inhibitor of the mTOR pathways [30, 31]. Melanoma-bearing C57BL/6J mice treated intraperitoneally with rapamycin in combination with metformin showed a significant reduction in the number of metastatic lung nodules compared with that in untreated control mice and mice treated with each drug separately (Figure 8A). Next, we similarly evaluated sitagliptin (Januvia^®^), which is a selective inhibitor of dipeptidyl peptidase 4 (DPP-4), and is used as monotherapy or in association with other anti-diabetic drugs (i.e. metformin) in the treatment of T2D. Treatment of melanoma-bearing C57BL/6J mice with metformin or sitagliptin resulted in a significant reduction in the number of metastatic lung nodules compared with the number of nodules in control mice (Figure 8B). Treatment with metformin and sitagliptin together showed a greater reduction in the number of metastatic lung nodules than did treatment with metformin or sitagliptin alone (Figure 8B).

Several reports have shown that metformin and sitagliptin exert an “off-target” effect on T cells [32]. No significant difference in the number of metastatic lung nodules was observed between metformin/sitagliptin-treated mice and the untreated control group when the experiment was performed on *Rag1*^−/−^ mice (data not shown). Overall, these results indicate that the anti-diabetic metformin and sitagliptin exert an anti-tumor immune response by affecting T cell performance.

### Metformin combined with sitagliptin exerts a strong effect in the BRAF/PTEN mouse model

Mutations in the serine/threonine kinase BRAF are the most common genetic alterations in human melanoma; however, B16F10 murine melanoma cells do not present this mutation [33]. We therefore tested whether a metformin/sitagliptin combination could also exert an effect in a murine tumor model with conditional melanocyte-specific expression of BRAF (V600E). In this melanoma pre-clinical model, Tyr:CreER:Braf^CA^:Pten^lox^/^lox^ (BRAF/PTEN), topical application of 4-hydroxytamoxifen (4-HT) to the skin induces an active Braf V600E mutation and Pten deletion in melanocytes [34, 35]. Five days after 4-HT applications to BRAF/PTEN mice, we initiated treatment with metformin/sitagliptin by gavage for 40 consecutive days. Metformin/sitagliptin-treated BRAF/PTEN mice showed a significant increase in median survival compared to that reported for untreated BRAF/PTEN mice (Figure 8C). These results confirm that the effect of the metformin/sitagliptin combination on B16F10 cells was similar to the effect on melanoma cells harboring the BRAF mutation.

## DISCUSSION

The results of this study showed that metformin can not only induce the cell death process but also exert a strong immunomodulatory effect, which can be enhanced by combination with other metabolism-targeting drugs to treat melanoma metastases. Recent studies have demonstrated that metformin acts on the immune system in various disease therapies [14, 36, 37]. In this, we showed that treatment of human and murine melanoma cells with metformin *in vitro* induces cell death through pleiotropic mechanisms.

Molecules involved in autophagy, such as ATG17, Beclin-1, and cathepsin B, or molecules associated with apoptosis, such as Bax, HIF-1α, and SOCS3 were up-regulated by metformin treatment. These data are consistent with those reported in previous studies on melanoma and other tumors [38–40]. However, in this study, metformin was also found to induce a caspase-independent death pathway as well as RIPK1, a key molecule in necroptosis. The role of RIPK1 in autophagy is not yet clearly understood, although a recent report suggested that RIPK1 represses basal autophagy via ERK-mediated phosphorylation of transcription factor EB [41]. Castration-resistant prostate cancer cells treated with metformin and simvastatin were shown to induce Ripk1- and Ripk3-dependent necrosis, suggesting that these necroptosis molecules may be important in the antitumor effects mediated by metformin [42]. The caspase-independent effects demonstrated here were also observed in cardiomyocytes [43]. The *in vitro* migration of human melanoma cells significantly decreased in the presence of metformin, at a dose that did not affect the viability of cells. This suggests that the drug, even at suboptimal doses, could affect tumor development. Recently, Liang et al. showed that 2.5- and 5-mM doses of metformin up-regulated the expression of E-cadherin in B16F10 cells, inhibiting cell motility, migration, and invasion, in a dose-dependent manner [44]. We know that primary cells derived from different patients can behave differently in culture conditions, and the expression of genes important to metformin's effect could be dramatically different between cells lines with distinct origins. Sensitivity to metformin may also be mediated by expression levels of the organic cation transporters (OCT1, OCT2, and OCT3). OCT1 is primarily responsible for metformin transport into cells, and polymorphisms in the gene encoding OCT1 that affect hepatic uptake of metformin *in vitro* have been associated with decreased efficacy in patients [45]. However, there is little information on the expression of OCT1 in human tumors. Thus, expression of these receptors, among others factors, may contribute to metformin resistance in different patient cell lines.

However, the direct effects of metformin on melanoma cells alone cannot fully account for the potent anti-metastatic activity that was observed *in vivo* in B16F10 melanoma model. In fact, our studies with NSG mice showed that immune system activation is necessary for the anti-metastatic activity of metformin, and metformin treatment induces a local and systemic cytokine response. This is in line with a recent report that the antitumor effect of metformin may be mediated mainly via the immune system [46]. The study demonstrated that metformin can increase the number of CD8^+^ T cells infiltrating tumors and protect cells from apoptosis and depletion, as characterized by decreased production of IL-2, TNF-α, and IFN-γ [46]. In this study, we observed that metformin decreased the number of Gr1^+^CD11b^+^ cells in the spleen of tumor-bearing mice. These cells are phenotypically similar to MDSCs, which are important suppressors of the tumor immune response and include two major subsets: i) CD11b^+^Ly6G^+,^ which are immature granulocyte cells, and ii) CD11b^+^Ly6C^+^, which are immature monocytic cells [47].

Although a systemic increase in IFN-γ and TNF-α was observed, IL-10 expression increased in the tumor-bearing mice treated with metformin. Modulation of these opposing cytokines by metformin may reflect the high number of tumor-infiltrating lymphocytes (TIL) in treated mice. Our results suggested an important role for T lymphocytes in the protective immune response induced by metformin treatment of B16F10 melanoma-bearing mice. CD8^+^CD69^+^ and CD8^+^ effector-memory increased in metformin-treated mice, and CD8^+^ depletion reduced the anti-metastatic effect of metformin. CD4^+^ T cells are also important to the anti-metastatic effect of metformin in our model. The effect of metformin was attenuated in CD4^+/−^ knockout mice and in CD4^+^ depletion assays. Recent reports have shown that CD4^+^ T cells have different mechanisms to enhance effector and memory CD8^+^ T cell functions, which may explain this result [48, 49]. We therefore believe that a possible interpretation of our results is the requirement of CD4^+^ Th cells for activation of CD8^+^ CTLs [50]. Furthermore, several findings indicate a role for CD4^+^ T cells in antitumor responses beyond providing support for CD8^+^ CTLs [48, 51]. Worth to remember that the lower number of lung nodules in RAG1^−/−^ mice in the B16F10 metastatic model has already been observed [52]. These authors reported that mice lacking T cells, or depleted of CD4^+^ and CD8^+^ T cells, were resistant to pulmonary B16 growth, but not to subcutaneous growth.

In the lungs of tumor-bearing mice, it was observed a significant increase in the numbers of CD4^+^Foxp3^+^IL-10^+^ and a significant decrease in ROR-γ^+^IL-17A^+^CD4^+^ T cells T cells in metformin-treated and protected animals, phenotypically similar to Treg and Th17 cells, respectively. Several studies have reported that metformin can ameliorate inflammatory and autoimmune diseases by regulating the Th17/Treg balance [15, 29, 53]. Increased numbers of Treg cells after treatment with metformin was observed for RL male 1 leukemia cells [46]. Th17 down-regulation by metformin has also been demonstrated in a hepatocellular carcinoma model associated with a decrease in IL-22 secretion, as well as a transient increase in Treg cells in a leukemia solid tumor model [14, 46]. Although Tregs are associated with tumor development, one study demonstrated that IL-10 from Tregs induced by type I IFN is necessary to negatively regulate Th17 inflammation in the tumor microenvironment [54]. In addition, evaluation of bacterial and viral murine models demonstrated that Tregs induce high-avidity CD8^+^ T cells in primary responses and promote maturation of CD8^+^ T cells in the resolution phase of infection [49, 55]. Despite this, it is necessary a better understanding about the mitochondrial impact of metformin on T cells present in tumor microenvironment. Mitochondria is the first intracellular target of metformin where this drug inhibits the complex I of the electron transport chain, leading to a drop-in energy load [56, 57]. The specific molecular mechanisms involved in inhibition of the mitochondrial complex I remain obscure. It has been reported that there is an interaction between biguanides and mitochondrial copper ions, and this interaction seems to be crucial for the metabolic effects of metformin [58]. Previous research has shown that this effect in the mitochondria is important for the antitumoral activity of metformin [59]. Also, recently it was reported that mitochondrial activation on T cells increases CTL function against tumor [60, 61].

Several agents targeting cancer metabolism have potential therapeutic value [62]. However, only a few of these drugs have been evaluated in relation to their effects on others cells in the tumor microenvironment, particularly for immune system cells. Metformin, rapamycin, and sitagliptin are clinical drugs with well-known metabolic features. Some studies have shown both anticancer action of and modulation of immune cells by these agents [6, 63–65]. Rapamycin has an anti-metastatic effect and can influence T cell biology via mTOR modulation [63, 66]. It may also cooperate with anti-CTLA-4 to increase CD8^+^ T cell memory [66]. Our findings suggest that a metformin/sitagliptin combination may contribute to treating melanoma with different driver mutations, including melanoma with or without a BRAF (V600E) mutation. Sitagliptin, a specific DPP-4 inhibitor, has an important effect on CD4^+^ T cells in addition to its anti-diabetic effects, and a recent report showed that DPP4 inhibition could increase lymphocyte trafficking, thereby improving antitumor immunity by CXCL10 preservation [32, 67]. Drug combinations targeting T cell immunometabolism could prevent allograft rejection [30]. Several clinical trials are currently using metformin alone or in combination with others agents, including immune checkpoint inhibitors [68].

In this study, we report that the antitumor effect of metformin besides the induction of cell death, with distinct features, rely on immune cells in special T cells present in the tumor microenvironment. Moreover, we offer evidence supporting that metformin combined with other clinical metabolic drug targets, such as mTOR and DPP4 inhibitors, could be repurposing as a strategy for treating cancer.

## MATERIALS AND METHODS

### Ethics statement

The investigation was conducted in accordance with ethical standards and national and international guidelines. All animal experiments were approved by the Animal Experimentation Ethics Committee of the Institute of Biomedical Sciences of the University of São Paulo (CEUA number: 017-2015).

### Cell lines

All experiments were performed with murine melanoma B16F10 cells, deposited at BCRJ (Rio de Janeiro Cell Bank, Brazil) code 0046. The melanoma patient cell lineages were kindly provided by M.D. Ph.D. Débora C.P. Silva (Ludwig Institute for Cancer Research, São Paulo). The cell lines MEL11, MEL26, and MEL28 [69] were cultured in RPMI 1640 medium (Invitrogen, Carlsbad, CA, USA) supplemented with 24 mM sodium bicarbonate, 10 mM HEPES (Sigma-Aldrich, St. Louis, MO, USA), 40 mg/L gentamicin (Gibco, Carlsbad, CA, USA), pH 7.2, and 10% fetal bovine serum (FBS, Gibco), and maintained frozen in liquid nitrogen. After thawing a frozen aliquot, cells were used until a maximum of 4 growth cycles.

### Animals and *in vivo* assays

C57BL/6, RAG1^−/−^, CD4^−/−^, CD8^−/−^, and Foxp3-eGFP mice, 6–8 weeks old, were purchased from our Isogenic Breeding Unit (Immunology Department, Biomedical Science Institute, University of São Paulo). NSG mice (NOD scid gamma or NOD.Cg-Prkdcscid Il2rgtm1Wjl/SzJ) were purchased from the Center for Development of Experimental Models (CEDEME), at UNIFESP. Mice were injected intravenously via the caudal vein with 5×10^5^ B16F10 melanoma cells (0.1 mL/mouse). Three days after tumor cell challenge, metformin (Merck, Kenilworth, NJ, USA; 500 mg/kg/day), rapamycin (Sigma-Aldrich; 1.5 mg/kg), sitagliptin (Januvia, Merck; 50 mg/kg), or PBS was administered daily by gavage until the 14^th^ experimental day. Lung metastatic nodules were counted using an inverted microscope on the 15^th^ day. For depletion assays, C57Bl/6J mice were treated by intraperitoneal injection with monoclonal antibodies, clones GK1.5 to deplete CD4^+^ or clones PC61 to deplete CD25^+^. GK1.5 (500 μg/dose) to deplete CD4^+^ was inoculated 72 and 24 h before i.v. tumor cell challenge; P61 (500 μg/dose) was inoculated 24 h prior, and also 4 and 7 days after tumor cell challenge. Tyr-CreERt: BrafCA:Ptenlox/lox, a transgenic murine model of BRAF V600 mutation in a heterozygous genetic background, (Jackson laboratory, Bar Harbor, ME, USA) was intradermally injected (i.d.) with 4-hydroxytamoxifen (4-HT) in the back for mutation induction. Treatment with metformin and sitaglipitin iniciated on the 5^th^ day after 4-HT inoculation.

### Cell viability assay

B16F10 and melanoma patient tumor cells were plated onto 96-well plates (5×10^4^ cells/mL) and treated with different concentrations of metformin (Merck) for 24, 48, or 72 h. Viable cells were determined by the MTT assay (Sigma-Aldrich). We also assessed cellular viability by measuring extracellular lactate dehydrogenase (LDH) in culture media using a colorimetric method (Labtest, Vista Alegre, MG, Brazil). Where indicated, cells were incubated with caspase-family inhibitor Z-VAD fluoromethylketone (Z-VAD-FMK, Biovision, USA) 2mM, caspase-1 inhibitor (Z-YVAD-FMK, Biovision, USA) and the necroptosis inhibitor necrostatin-1 (nec-1, Sigma-Aldrich) at a concentration of 10μM.

### Migration wound-healing assay

Tumor melanoma cells were plated onto 6-well plates (1 × 10^6^ cells/mL) in triplicate. After cell attachment and growth to a confluent monolayer (higher than 80%), the medium was pipetted out and replaced by PBS, and one scratch wound was made with a P1000 tip (Thermo Fisher Scientific, Waltham, MA, USA) in each well. PBS was pipetted out, serum-free RPMI medium with metformin was added to each well, and cells were incubated for 3, 7, and 24 h. The cell migration distance was determined by measuring the width of the wound and subtracting this value from the initial half-width value of the wound.

### Gene profiles and qPCR

Total RNA was isolated from the spleen and lung samples that were snap-frozen in liquid nitrogen or B16F10 cultures treated with or without metformin by using TRIzol reagent (Invitrogen) according to the manufacturer's instructions. RNA concentrations were determined by spectrophotometry readings at 260 nm. First-strand cDNA was synthesized using MMLV reverse transcriptase (Promega, Madison, WI, USA). RT-PCR was performed using the Taqman real-time PCR assay (Applied Biosystems, Foster City, CA USA) for the following molecules: HPRT (Mm00446968_m1), TNF-α (Mm00443258_m1), IL-6 (Mm004461690_m1), SOCS3 (Mm00545913_s1), IFNγ(Mm00801778_m1), IL-10 (Mm439616_m1), FOXP3 (Mm00475156_m1), RORc (Mm01261022_m1), HIF1α (00468878_m1), IL-1β (Mm00434228_m1), IL-17 (Mm00439619_m1), and Beclin-1 (Mm01265461_m1). Cycling conditions were as follows: Ten minutes at 95°C followed by 45 cycles at 20 s each at 95°C, 20 s at 58°C, and 20 s at 72°C. Sequence Detection Software 1.9 (SDS) was used for analysis. mRNA expression was normalized to HPRT expression, calculated with the following equation: Relative expression level of the target mRNA = 2−ΔΔCt. The cell death PCR array (PAMM-212Z, SABiosciences, Frederick, MD, USA), with primers for 84 pathway-specific genes, was performed according to the manufacturer's instructions.

### Flow cytometry

Foxp3-GFP^+^ cells and T subset cells were analyzed by multicolor flow cytometry. The monoclonal antibodies used were CD3, CD4, CD8, CD44, CD62L (BD Biosciences, Franklin Lakes, NJ, USA), CD11b, CD11c, F4/80, Ly6G, and Ly6C (BioLegend, San Diego, CA, USA). For activation, single-cell suspensions of tumor cells or spleen were resuspended in RPMI containing 100 ng/ml PMA (Sigma-Aldrich), 1 μg/ml ionomycin (Sigma-Aldrich), and brefeldin A (Sigma-Aldrich) for 4 hours at 37°C. Intracellular staining was performed with FITC-conjugated anti-ROR-γ, anti-Foxp3, anti-IL10 anti-IL-17 and anti-IFN-γ (Biolegend). Annexin-V/7AAD (BioLegend, San Diego, CA, USA) staining was performed after B16F10 treatment with metformin (10-20 mM). The fluorescence intensity of fluorochrome-labeled cells was measured by flow cytometry (FACSCanto II, BD Biosciences). FACSDiva software was used for calculating cell numbers, and data analysis was performed by FlowJo (FlowJo LLC, Ashland, OR, USA).

### Measurement of intracellular ROS

Intracellular accumulation of ROS was monitored using 2,7-dichlorofluorescein diacetate (DCF-DA, Thermo Fisher Scientific, Waltham, MA, USA) or MitoSOX (Molecular Probes, OR, USA). B16F10 cells were seeded into 96-well plates and treated with different concentrations of metformin (Merck) for 24 h. The medium was removed and cells were washed and incubated with DCF-DA (5 μM) or MitoSOX (5 μM) for 30 min. Then, cells were washed and analyzed by flow cytometry.

### Seahorse extracellular flux

Seahorse analysis experiments were performed as previously described [61]. In brief, B16F10 cells were plated and treated with 10 mM of metformin 1 day prior to the seahorse experiment. Cells were allowed to equilibrate at 37°C for 30 min prior to starting the assay. Metformin (50 μM), oligomycin (ATPase inhibitor, 0.5 μM), and FCCP (0.2 μM) were injected and OCR (pmol/min) was measured.

### Cytokine evaluation

Cytokine concentrations were measured in the serum, spleen, and lung homogenates, then collected on the 15^th^ day after tumor cell inoculation. Lung homogenates were prepared by digesting lung tissues with 1U/mL collagenase IV (Sigma-Aldrich) at 37°C for 30 min. After centrifugation, the supernatants were stored at −80°C. IFN-γ, TNF-α, IL-10, IL-17, IL-1β, IL-6, and adiponectin were quantified by sandwich ELISA, following the manufacturer's instructions (R&D Systems, Minneapolis, MN, USA).

### Statistical analysis

Statistical analysis of experimental and control data was conducted by a Student *t*-test or two-way ANOVA with a Bonferroni post hoc test for multiple comparisons. In all studies, a value of *p*<0.05 was considered statistically significant.

## SUPPLEMENTARY MATERIALS FIGURES



## Abbreviations

T2D: Type 2 diabetes
AMPK: AMP-activated kinase
IL: interleukin
RIPK1: receptor-interacting serine/threonine-protein kinase 1
OCR: oxygen consumption rate
LDH: lactate dehydrogenase
