# Supplementary material for: Metformin exerts antitumor activity via induction of multiple death pathways in tumor cells and activation of a protective immune response
Source: Oncotarget. 2018 May 25;9(40):25808–25. doi: 10.18632/oncotarget.25380 (PMC5995253; doi:10.18632/oncotarget.25380)
Supplement: Supplementary file 1 [file oncotarget-09-25808-s001.pdf]

Metformin exerts antitumor activity via induction of multiple death pathways in tumor cells and activation of a protective immune response

SUPPLEMENTARY MATERIALS

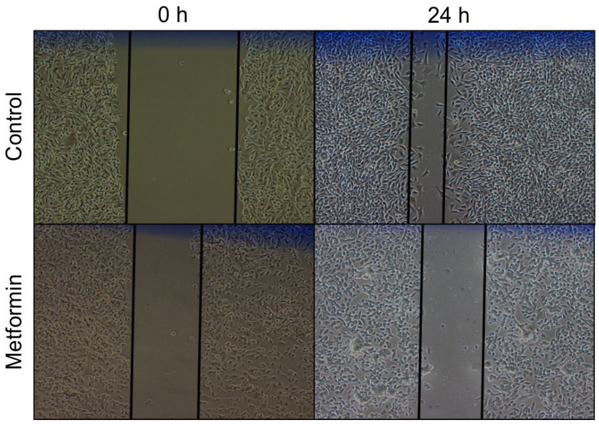

Supplementary Figure 1: Effect of metformin on human melanoma cell migration. MEL-11 cell migration was determined by measuring the width of the wound at baseline (0 h), and 24 h after incubation with 5mM metformin.

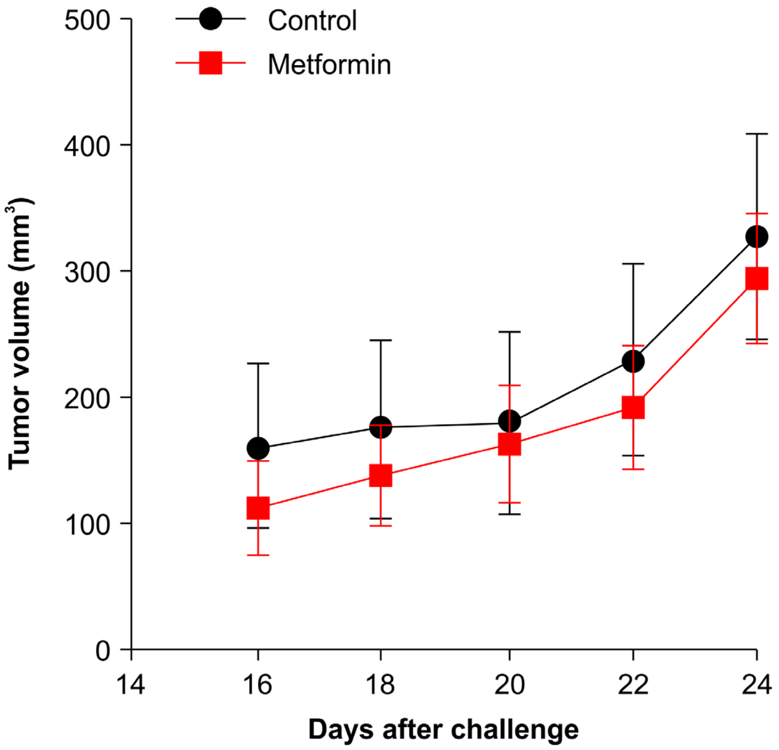

Supplementary Figure 2: Antitumor activity of metformin in subcutaneously grafted melanoma. C57BL/6 mice were subcutaneously challenged with B16F10 cells, and treated by gavage with 500 mg/kg metformin or phosphate-buffered saline daily from day 3 after challenge.
